# Neuropeptide Y and peptide YY protect from weight loss caused by Bacille Calmette–Guérin in mice

**DOI:** 10.1111/bph.12354

**Published:** 2013-10-15

**Authors:** Evelin Painsipp, Martin J Köfer, Aitak Farzi, Ulrich S Dischinger, Frank Sinner, Herbert Herzog, Peter Holzer

**Affiliations:** 1 Research Unit of Translational Neurogastroenterology, Institute of Experimental and Clinical Pharmacology, Medical University of GrazGraz, Austria; 2 Health – Institute for Biomedicine and Health Sciences, Joanneum ResearchGraz, Austria; 3 Neurobiology Research Program, Garvan Institute of Medical ResearchSydney, Australia

**Keywords:** Bacille Calmette–Guérin, corticosterone, exploration, food intake, immune stimulation, IL-6, locomotion, neuropeptide Y, peptide YY, sickness behaviour

## Abstract

**Background and Purpose:**

Immune challenge of mice with Bacille Calmette–Guérin (BCG) has been reported to cause transient weight loss and a behavioural sickness response. Although BCG-induced depression involves the kynurenine pathway, weight loss occurs independently of this factor. Because neuropeptide Y (NPY) and peptide YY (PYY) are involved in the regulation of food intake, we hypothesized that they play a role in the BCG-induced weight loss.

**Experimental Approach:**

Male wild-type, PYY knockout (PYY−/−), NPY knockout (NPY−/−) and NPY−/−;PYY−/− double knockout mice were injected with vehicle or BCG (approximately 10^8^ colony-forming units per mouse), and their weight, locomotion, exploration and ingestion were recorded for 2 weeks post-treatment.

**Key Results:**

Deletion of PYY and NPY aggravated the BCG-induced loss of body weight, which was most pronounced in NPY−/−;PYY−/− mice (maximum loss: 15%). The weight loss in NPY−/−;PYY−/− mice did not normalize during the 2 week observation period. BCG suppressed the circadian pattern of locomotion, exploration and food intake. However, these changes took a different time course than the prolonged weight loss caused by BCG in NPY−/−;PYY−/− mice. The effect of BCG to increase circulating IL-6 (measured 16 days post-treatment) remained unaltered by knockout of PYY, NPY or NPY plus PYY.

**Conclusions and Implications:**

These data show that NPY and PYY are both required to protect from the action of BCG-evoked immune challenge to cause prolonged weight loss and disturb energy balance. The findings attest to an important role of NPY and PYY in orchestrating homeostatic reactions to infection and immune stimulation.

## Introduction

Immune challenge of CD1 mice with Bacille Calmette–Guérin (BCG) induces an acute episode of sickness behaviour characterized by weight loss, reduced locomotion and fever (Moreau *et al*., 2008). This transient sickness response to BCG is followed by changes in affective behaviour (Moreau *et al*., 2008; O'Connor *et al*., 2009a,b2009b). The BCG-induced depression-like behaviour depends on the formation of TNF-α and IFN-γ that cause an up-regulation of indoleamine 2,3-dioxygenase and thus enforce the kynurenine pathway of tryptophan utilization (Moreau *et al*., 2008; O'Connor *et al*., 2009a,b2009b). In contrast, symptoms of the acute sickness response to BCG such as weight loss occur independently of these signalling pathways (Moreau *et al*., 2008; O'Connor *et al*., 2009a,b2009b).

We have previously shown that the neuropeptide Y (NPY) system plays a protective role against the behavioural responses to LPS (Painsipp *et al*., 2008; 2010,). This bacterial cell wall constituent stimulates the formation of cytokines and evokes a transient sickness response (Dantzer *et al*., 2008), and under particular circumstances, promotes the development of depression-like behaviour (Painsipp *et al*., 2010). Mice in which the Y2 receptor (Alexander *et al*., 2011) has been deleted are particularly sensitive to the acute effect of LPS to attenuate locomotion and social interaction (Painsipp *et al*., 2008). These observations led us to hypothesize that the NPY system regulates neurobiological responses not only to LPS but also to live mycobacteria of the BCG type. This question was addressed by examining some of the behavioural effects of BCG in peptide YY knockout (PYY−/−), NPY knockout (NPY−/−) and NPY plus PYY double knockout (NPY−/−;PYY−/−) mice.

An implication of NPY and PYY in the behavioural manifestations of immune challenge is suggested by many properties of this peptide family. NPY is involved in the cerebral regulation of food intake, cognition, anxiety, mood and stress resilience (Heilig, 2004; Lin *et al*., 2004; McGowan and Bloom, 2004; Ueno *et al*., 2008; Morales-Medina *et al*., 2010), and these implications are borne out by a distinct phenotype of NPY−/− mice (Bannon *et al*., 2000; Lin *et al*., 2004; Karl *et al*., 2008; Painsipp *et al*., 2011; Forbes *et al*., 2012). PYY is a gut hormone, and the major circulating form of this peptide, PYY(3–36), is thought to be a satiety signal, inhibiting food intake via stimulation of auto-inhibitory Y2 receptors in the arcuate nucleus of the hypothalamus (McGowan and Bloom, 2004; Ueno *et al*., 2008). Consequently, PYY−/− and NPY−/− mice exhibit appreciable alterations in feeding behaviour (Bannon *et al*., 2000; Lin *et al*., 2004; Batterham *et al*., 2006; Boey *et al*., 2006; Karl *et al*., 2008; Edelsbrunner *et al*., 2009; Forbes *et al*., 2012).

Because the cell wall of BCG is, like that of gram-positive bacteria, deficient in LPS, the effects of BCG and LPS on the immune system are likely to involve differential mechanisms. Against this background, the current study set out to address three specific issues. The first aim was to establish whether the sickness response to BCG as studied in CD1 mice (Moreau *et al*., 2008; O'Connor *et al*., 2009a,b2009b) can be reproduced in C57BL/6 mice, given that the wild-type (WT), PYY−/−, NPY−/− and NPY−/−;PYY−/− mice have a C57BL/6:129/SvJ (1:1) background. It was therefore examined whether BCG causes weight loss and fever in C57BL/6 mice. In addition, it was investigated whether BCG enhances anxiety in the open field (OF) test and stimulates the hypothalamic-pituitary-adrenal (HPA) axis as deduced from a rise of circulating corticosterone.

The second aim was to explore whether deletion of NPY and/or PYY alters the ability of BCG to cause immune stimulation and weight loss. To this end, we studied the effect of BCG on body weight (BW) and circulating levels of IL-6 in WT, PYY−/−, NPY−/− and NPY−/−;PYY−/− mice within a time frame of 2 weeks post-treatment.

The third aim was to analyse the BCG-evoked weight loss, which was most pronounced in NPY−/−;PYY−/− mice, by recording the circadian patterns of locomotion, exploration, water and food intake over a period of 2 weeks post-treatment.

## Methods

### Experimental animals

The study was conducted with age-matched, 3-to 5-month-old, male mice of five different genotypes: WT (*n* = 17), PYY−/− (*n* = 8), NPY−/− (*n* = 8) and NPY−/−;PYY−/− (*n* = 32) mice, all on a mixed C57BL/6:129/SvJ (1:1) background, as well as C57BL/6N mice (*n* = 38). Germ line PYY−/− mice (Boey *et al*., 2006) as well as germ line NPY−/− mice (Karl *et al*., 2008) in which the entire coding sequence including the initiation start codon was removed were generated as reported. Double knockout (NPY−/−;PYY−/−) mice were generated by crossing the single-gene knockout strains and in a second step by crossing the double heterozygous mice (Zhang *et al*., 2012). The presence or deletion of PYY and/or NPY was verified by PCR (Boey *et al*., 2006; Karl *et al*., 2008). Homozygous male and female WT, NPY−/−, PYY−/− and NPY−/−;PYY−/− mice were obtained from the Neurobiology Research Program of the Garvan Institute of Medical Research (Sydney, Australia) via the Institute of Pharmacology of the Medical University of Innsbruck (Innsbruck, Austria). No more than three generations of these homozygous animals were bred at the Institute of Experimental and Clinical Pharmacology of the Medical University of Graz (Graz, Austria). The C57BL/6N mice were obtained from Charles River Laboratories (Sulzfeld, Germany).

The animals were housed in groups of four in cages of size IIL (length × width × height = 26.0 × 20.5 × 14.0 cm) under controlled temperature (set point 22°C), relative air humidity (set point 50%) and light conditions (lights on at 0600 h, lights off at 1800 h). Tap water and standard laboratory chow were provided *ad libitum* throughout the study.

The experimental procedures and number of animals used in this study were approved by an ethical committee at the Federal Ministry of Science and Research of the Republic of Austria and conducted according to the Animal Experimentation Law in Austria and the Directive of the European Communities Council of 24 November 1986 (86/609/EEC). The experiments were conducted in such a way that the number of animals used and their suffering was minimized. The ARRIVE guidelines (Kilkenny *et al*., 2010; McGrath *et al*., 2010) were followed in designing the study and reporting the experiments.

### Administration of BCG

A fresh solution of BCG (ImmuCyst®, Sanofi Pasteur SA, Lyon, France) was made on the day of injection. To this end, the freeze-dried BCG content [equivalent to approximately 10^9^ colony-forming units (CFU)] of an ImmuCyst vial was suspended in 3 mL of the diluent provided by the manufacturer, yielding a suspension of approximately 3.3 × 10^8^ CFU·mL^−1^. The BCG suspension was administered i.p. at an approximate dose of 1 × 10^8^ CFU per mouse (O'Connor *et al*., 2009a) in an injection volume of 0.3 mL per mouse. The diluent (0.85% sodium chloride; 0.025% Tween 80, Sigma-Aldrich, Vienna, Austria; 0.06% sodium dihydrogen phosphate; and 0.25% disodium hydrogen phosphate) was used as vehicle (VEH) treatment.

### Experimental protocols

The experiments were started after the animals had become familiar with the institutional animal house over the course of at least 3 weeks. Prior to the experiments, the mice were allowed to adapt to the test room (set points 22°C and 50% relative air humidity, lights on at 0600 h, lights off at 1800 h, maximal light intensity 100 lux) for at least 5 days. Three different protocols were used.

Protocol 1 was used to conduct a pilot study to confirm the activity of BCG to elicit a sickness response and to stimulate the HPA axis in five groups of C57BL/6N mice. For this purpose, the effect of VEH (*n* = 8) and BCG (*n* = 8) on BW and rectal temperature was evaluated immediately before treatment and 1, 3 and 7 days post-injection. In addition, the animals were subjected to the OF test on day 7 post-injection. In a separate experiment, the plasma concentration of corticosterone in trunk blood was measured in VEH-treated (*n* = 8) and BCG-treated (*n* = 8) mice 1 day post-injection and compared with that in untreated mice (*n* = 6).

In protocol 2, the effect of VEH and BCG on BW and circulating levels of IL-6 was investigated in WT (*n* = 8), PYY−/− (*n* = 8), NPY−/− (*n* = 8) and NPY−/−;PYY−/− (*n* = 8) mice. BW was recorded immediately before treatment and 1, 3, 7, 12 and 15 days post-injection. The concentrations of IL-6 in trunk blood were estimated 16 days post-injection.

In protocol 3, the LabMaster system (TSE Systems, Bad Homburg, Germany) was employed to analyse the effect of VEH and BCG on the circadian pattern of locomotion, exploration, drinking and feeding in singly housed WT (*n* = 9) and NPY−/−;PYY−/− (*n* = 24) mice. The single housing protocol was started 7 days before injection of VEH or BCG. During this time, the animals were habituated to the drinking bottles used in the LabMaster system. Three days before injection, the mice were placed in the test cages of the LabMaster system and maintained there until 14 days post-injection.

### Body temperature

The rectal temperature was measured with a digital thermometer (BAT-12, Physitemp Instruments, Clifton, NJ, USA) equipped with a rectal probe for mice.

### OF test

The OF consisted of a box (50 × 50 × 30 cm) that was made of opaque grey plastic and illuminated by 80 lux at floor level. The ground area of the box was divided into a 36 × 36 cm central area and the surrounding border zone (Painsipp *et al*., 2011). The mice were placed individually in the centre of the OF, and their behaviour during a 5 min test period was tracked by a video camera positioned above the centre of the OF and recorded with the software VideoMot2 (TSE Systems, Bad Homburg, Germany). This software was used to evaluate the time spent in the central area, the number of entries into the central area and the total distance travelled in the OF. A reduction of the central area time and/or the central area entries was interpreted as an increase in anxiety-like behaviour (Painsipp *et al*., 2011).

### Circulating corticosterone

Between 1000 and 1200 h, the mice were deeply anaesthetized with pentobarbitone (150 mg·kg^−1^ i.p.) before they were decapitated. Within 2 min after the injection of anaesthetic, trunk blood was collected into vials coated with EDTA (Greiner, Kremsmünster, Austria) kept on ice. Following centrifugation for 10 min at 4°C and 1200 × *g*, blood plasma was collected and stored at −70°C until assay. The plasma levels of corticosterone were determined with an enzyme immunoassay kit (Assay Designs, Ann Arbor, MI, USA). According to the manufacturer's specifications, the sensitivity of the assay is 27 pg·mL^−1^, and the intra-and inter-assay coefficient of variation amounts to 7.7 and 9.7% respectively.

### Circulating IL-6

A part of the blood plasma collected for determination of corticosterone was used for the assay of circulating IL-6. The plasma levels of IL-6 were determined with an enzyme immunoassay kit (Fluorokine MAP Mouse IL-6 Kit, R&D Systems, Minneapolis, MN, USA). According to the manufacturer's specifications, the sensitivity of the assay is 1.1 pg·mL^−1^, and the intra-and inter-assay coefficient of variation amounts to 4.0 and 7.4% respectively.

### LabMaster system

The circadian pattern of locomotion, exploration, drinking and feeding was assessed with the LabMaster system that consisted of six recording units, each unit comprising a test cage (type III, 42.0 × 26.5 × 15.0 cm, length × width × height), two external infrared frames and a cage lid fitted with two weight transducers (Edelsbrunner *et al*., 2009). These devices were connected to a personal computer that was used to collect and analyse the data with the LabMaster software. The system was configured such that 720 values of each test parameter were collected over a 12 h interval.

The two weight transducers were employed to quantify ingestive behaviour. To this end, a feeding bin filled with standard rodent chow (altromin 1324 FORTI, Altromin, Lage, Germany) and a drinking bottle filled with tap water were each attached to a transducer on the cage lid, and the animals were allowed to drink and feed *ad libitum*. Water and food intake over time were measured in milligrams and grams respectively. For data analysis, the amount of water and food ingested over select time intervals was normalized to the BW of the animals (Edelsbrunner *et al*., 2009).

For recording locomotion and exploration, the two external infrared frames were positioned in a horizontal manner above one another at a distance of 4.3 cm, with the lower frame being fixed 2.0 cm above the bedding floor. The bottom frame was used to record horizontal locomotion (ambulatory movements) of the mice, whereas the top frame served to record vertical movements (rearing, exploration). The measures of activity (locomotion, exploration) were derived from the light beam interruptions (counts) of the corresponding infrared frames (Edelsbrunner *et al*., 2009).

### Statistics

Statistical evaluation of the results was made with SPSS 16.0 (SPSS Inc., Chicago, IL, USA). The data obtained from different genotypes were analysed by Student's *t*-test or anova, as appropriate. The homogeneity of variances was assessed with the Levene test. In case of sphericity violations the Greenhouse–Geisser correction was applied. Post-anova analysis of group differences was performed with the Tukey's honestly significant difference test, when the variances were homogeneous, and with the Games–Howell test, when the variances were unequal. Probability values *P* ≤ 0.05 were regarded as statistically significant. All data are presented as means ± SEM, *n* referring to the number of mice in each group.

## Results

### BCG increased circulating corticosterone and induced sickness-related behaviour in C57BL/6N mice

**Figure 1:**
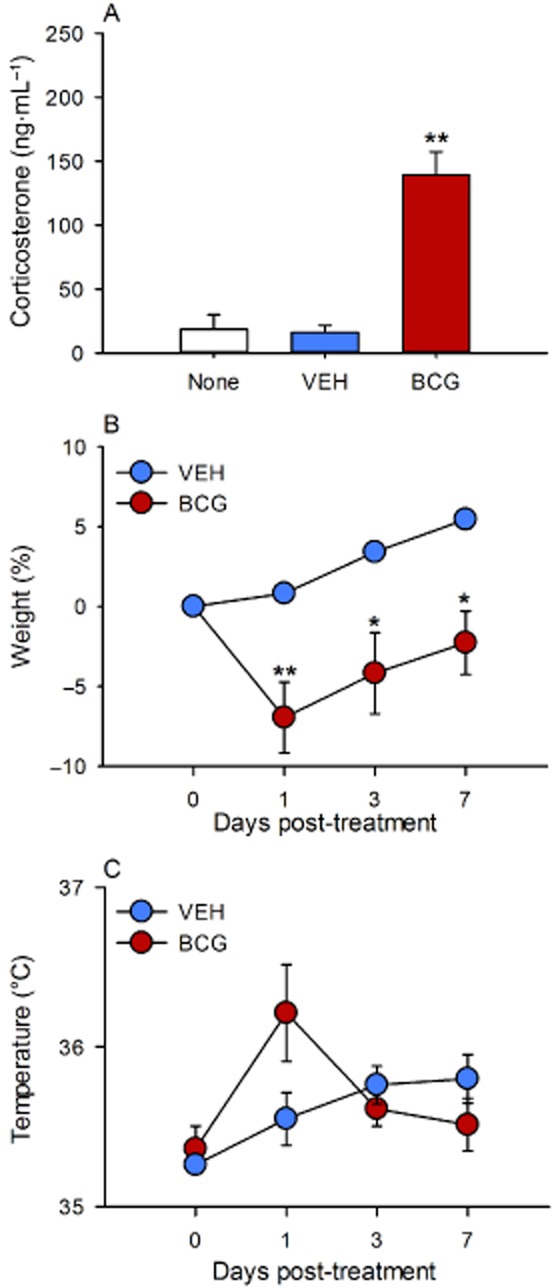
Effect of BCG (10^8^ CFU per mouse injected i.p.), relative to VEH, on circulating corticosterone (A), BW (B) and rectal temperature (C) in male C57BL/6N mice. Corticosterone was measured in trunk blood 1 day post-injection as well as in untreated mice. BW and rectal temperature were measured immediately before injection of VEH and BCG (day 0) and 1, 3 and 7 days post-injection. The values are means + SEM (A) and means ± SEM (B and C), *n* = 6–8. *P < 0.05, **P < 0.01 versus VEH.

I.p. injection of BCG to male C57BL/6N mice led to an approximately sevenfold increase in the plasma concentration of corticosterone as measured 1 day post-injection, relative to the corticosterone concentration measured 1 day after injection of VEH (Figure 1A). VEH did not seem to have a lasting effect on circulating corticosterone, given that the level of corticosterone in VEH-treated mice did not differ from those in untreated mice (Figure 1A).

A separate experiment showed that administration of BCG caused an approximately 7% reduction of BW 1 day post-injection (Figure 1B). Thereafter, BW began to recover but did not quite reach the pre-injection value during the 7 day post-treatment observation period. In contrast, VEH failed to reduce BW, which in VEH-treated animals continuously increased over the 7 day period post-injection (Figure 1B). Furthermore, BCG evoked a non-significant increase of rectal temperature as measured 1 day post-injection, which had disappeared by 3 days post-injection (Figure 1C). Thus, rectal temperature in BCG-treated mice had risen by 0.85 ± 0.28°C 1 day post-injection compared with a rise of 0.29 ± 0.20°C in VEH-treated mice.

**Figure 2:**
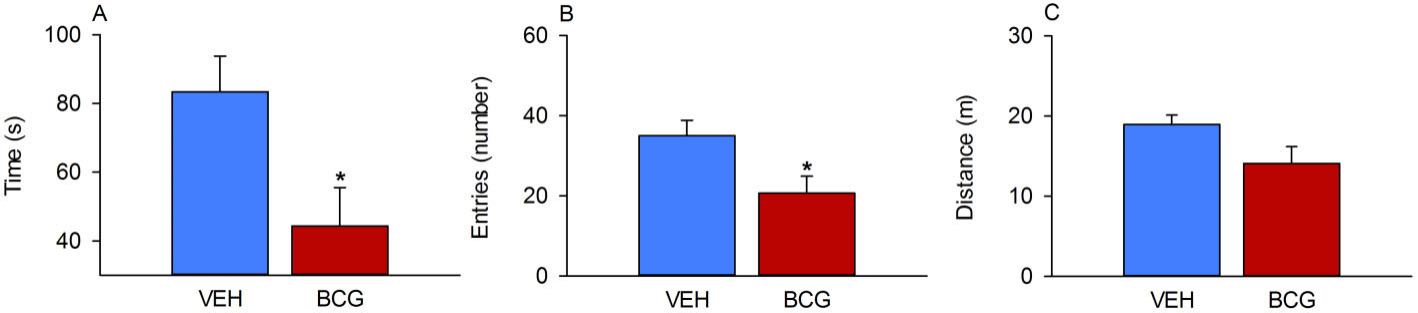
Effect of BCG (10^8^ CFU per mouse injected i.p.), relative to VEH, on behaviour in the OF as measured 7 days post-injection in male C57BL/6N mice. The graphs show the time spent in the central area (A), the number of entries into the central area (B) and the total distance travelled (C) during the 5 min test session. The values represent means + SEM, *n* = 8. *P < 0.05.

Seven days post-injection, the animals were subjected to the OF test to examine their locomotor/exploratory and anxiety-related behaviour. The time spent in the central area and the number of entries into the central area were considered as indices of anxiety. Analysis of the data showed that, relative to VEH, BCG enhanced anxiety-related behaviour as deduced from a significant reduction of the time spent in the central area and the number of entries into the central area (Figure 2A and B). The locomotor activity of BCG-treated mice as deduced from the total travelling distance was not significantly reduced relative to that seen in VEH-treated mice (Figure 2C).

### Deletion of NPY and PYY aggravated the BCG-induced loss of BW

**Figure 3:**
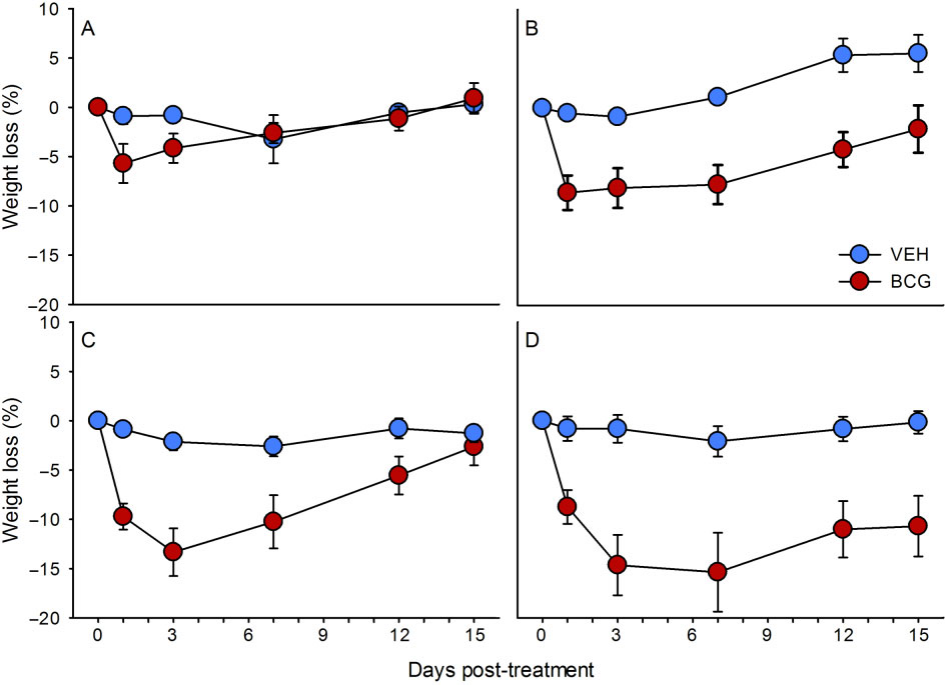
Effect of BCG (10^8^ CFU per mouse injected i.p.), relative to VEH, to reduce BW in male WT (A), PYY−/− (B), NPY−/− (C) and NPY−/−;PYY−/− (D) mice. The weight loss is expressed as a percentage of the BW measured immediately before injection of VEH and BCG (day 0) and shown for a 15 day post-injection period. The values are means ± SEM, *n* = 8.

Having established that BCG caused a protracted but transient decrease of BW in C57BL/6N mice, the time course of the VEH-and BCG-induced loss of BW was evaluated in WT, PYY−/−, NPY−/− and NPY−/−;PYY−/− mice for a period of 15 days post-injection. As shown in Figure 3A–D, BCG reduced BW in all genotypes to a larger extent than VEH. However, the magnitude of the BCG effect varied largely with the genotype and was particularly pronounced in NPY−/− and NPY−/−;PYY−/− mice (Figure 3C and D). In WT animals, BCG caused an approximately 6% weight loss as seen 1 day post-injection, an effect that was gone by day 7 post-injection when compared with VEH-treated WT mice (Figure 3A). The BCG-evoked weight loss in PYY−/− mice amounted to approximately 8.5% 1 day post-injection and, relative to the effect of VEH, did not reverse during the 15 day post-injection period because VEH-injected PYY−/− mice began to gain weight from day 7 post-injection onwards (Figure 3B). In NPY−/− mice, BCG lowered BW by approximately 13% as observed 3 days post-injection (Figure 3C). In comparison with the effect of VEH it took the NPY−/− mice 15 days to recover from the BCG-induced weight loss (Figure 3C). The most pronounced effect of BCG on BW was seen after injection of BCG to NPY−/−;PYY−/− mice (Figure 3D). In this genotype, the BCG-evoked weight loss proceeded for 7 days post-injection when it reached a maximum of about 15%. Thereafter, the BW began to recover but was still far from normal by day 15 post-injection (Figure 3D).

The statistical analysis of the data addressed both the maximal weight loss caused by BCG, relative to VEH, in the different genotypes and their time course. The two-way anova of the maximal decreases in BW seen in VEH-and BCG-treated mice disclosed that the weight loss varied with treatment [*F*_(1,55)_ = 29.77, *P* < 0.01] but not genotype [*F*_(3,55)_ = 1.96, *P* = 0.13], without a significant interaction between these factors [*F*_(3,55)_ = 2.20, *P* = 0.99]. Two-way anova for repeated measurements showed that the variation of weight with time and treatment differed in the four genotypes. In the WT mice, the weight varied with time [*F*_(1.85,25.92)_ = 3.30, *P* = 0.05] but not treatment [*F*_(1,14)_ = 2.34, *P* = 0.15] without a significant interaction between these factors [*F*_(1.85,25.92)_ = 2.55, *P* = 0.10]. In the PYY−/− mice, the weight depended on time [*F*_(3,42)_ = 11.83, *P* < 0.01] and treatment [*F*_(1,14)_ = 19.62, *P* < 0.01] with a significant interaction between these factors [*F*_(3,42)_ = 10.86, *P* < 0.01]. In the NPY−/− animals, the variation of weight with time [*F*_(1.56,21.79)_ = 21.48, *P* < 0.01] and treatment [*F*_(1,14)_ = 18.03, *P* < 0.01] was also significant, and there was likewise a significant interaction between these factors [*F*_(1.56,21.79)_ = 10.94, *P* < 0.01]. The same was true in NPY−/−;PYY−/− animals [variation with time: *F*_(1.58,20.6)_ = 9.99, *P* < 0.01; variation with treatment: *F*_(1,13)_ = 15.86, *P* < 0.01; interaction: *F*_(1.58,20.6)_ = 6.70, *P* < 0.01].

### Deletion of NPY and PYY did not alter the effect of BCG to increase circulating IL-6

**Figure 4:**
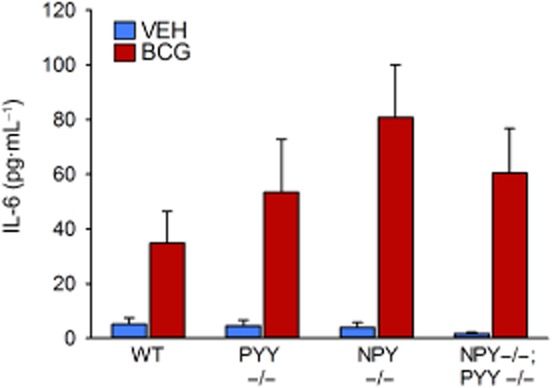
Effect of BCG (10^8^ CFU per mouse injected i.p.), relative to VEH, on plasma concentrations of IL-6 in male WT, PYY−/−, NPY−/− and NPY−/−;PYY−/− mice. IL-6 was measured in trunk blood 16 days post-injection. The values are means + SEM, *n* = 6–8.

The effect that BCG has on the circulating levels of IL-6 was measured 16 days post-injection. Relative to VEH, BCG enhanced the levels of circulating IL-6 in all genotypes investigated, that is, in WT, PYY−/−, NPY−/− and NPY−/−;PYY−/− mice (Figure 4). Although the nominal increase in the plasma levels of IL-6 was highest in NPY−/− mice, two-way anova showed that the IL-6 levels varied only with treatment [*F*_(2,47)_ = 16.64, *P* < 0.01] but not genotype [*F*_(3,47)_ = 1.05, *P* = 0.38], without a significant interaction between these factors [*F*_(3,47)_ = 1.20, *P* = 0.32].

### Deletion of NPY plus PYY aggravated the effect of BCG to depress circadian locomotion, exploration, and food and water intake

**Figure 5:**
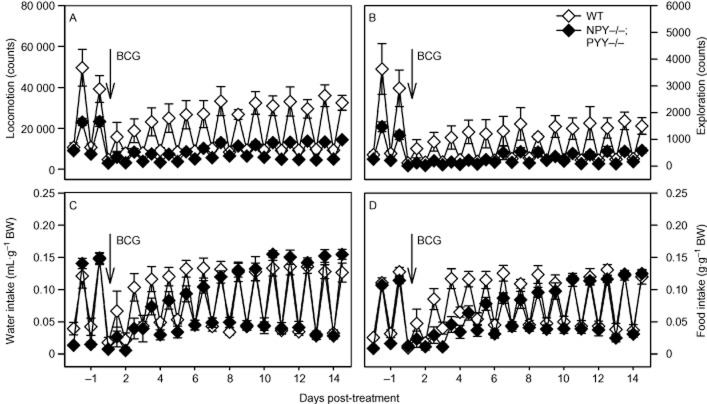
Effect of BCG (10^8^ CFU per mouse injected i.p.) on the circadian patterns of locomotion (A), exploration (B), drinking (C) and feeding (D) in male WT and NPY−/−;PYY−/− mice. BCG was injected as indicated. The graphs show the activity levels during the photophase (lower values) and scotophase (upper values) for 2 days before and 14 days after BCG injection. The values are means ± SEM, *n* = 9.

To analyse the pronounced effect of BCG on weight loss in NPY−/−;PYY−/− mice in more detail, the LabMaster system was employed to continuously record the locomotor (ambulatory), exploratory (rearing), drinking and feeding behaviour for 14 days post-injection. These parameters followed a characteristic circadian time course (Figure 5A–D). As was expected for nocturnal animals, the activity of mice was considerably higher during the scotophase than during the photophase.

Figure 5A–D illustrates that, before injection of BCG, there were appreciable differences in the basal circadian patterns of behaviour between the two genotypes, which have been reported previously (Edelsbrunner *et al*., 2009). Thus, the amplitude of the circadian locomotor and exploratory behaviour during the scotophase was considerably reduced in NPY−/−;PYY−/− mice, relative to WT animals, and drinking and feeding tended to be attenuated during the photophase (Figure 5A–D). A statistical comparison of the parameters measured during the photophase and scotophase immediately before injection of BCG is presented in Table 1.

**Table 1:** Locomotion, exploration, drinking and feeding in WT and NPY−/−;PYY−/− mice during the photo-and scotophase

Genotype	Circadian phase	Parameter	Value	*P*
WT	Photophase	Locomotion (counts)	10 668 ± 1732	
NPY−/−;PYY−/−	Photophase	Locomotion (counts)	7420 ± 582	=0.10
WT	Scotophase	Locomotion (counts)	39 235 ± 6609	
NPY−/−;PYY−/−	Scotophase	Locomotion (counts)	23 286 ± 2327	=0.04
WT	Photophase	Exploration (counts)	472 ± 112	
NPY−/−;PYY−/−	Photophase	Exploration (counts)	211 ± 68	=0.06
WT	Scotophase	Exploration (counts)	2910 ± 686	
NPY−/−;PYY−/−	Scotophase	Exploration (counts)	1155 ± 158	=0.02
WT	Photophase	Drinking (mL·g^−1^ BW)	0.0383 ± 0.0124	
NPY−/−;PYY−/−	Photophase	Drinking (mL·g^−1^ BW)	0.0149 ± 0.0042	=0.09
WT	Scotophase	Drinking (mL·g^−1^ BW)	0.1476 ± 0.0087	
NPY−/−;PYY−/−	Scotophase	Drinking (mL·g^−1^ BW)	0.1491 ± 0.0084	=0.90
WT	Photophase	Feeding (g·g^−1^ BW)	0.0316 ± 0.0061	
NPY−/−;PYY−/−	Photophase	Feeding (g·g^−1^ BW)	0.0163 ± 0.0033	=0.04
WT	Scotophase	Feeding (g·g^−1^ BW)	0.1278 ± 0.0066	
NPY−/−;PYY−/−	Scotophase	Feeding (g·g^−1^ BW)	0.1144 ± 0.0054	=0.13

The locomotor, explorative and ingestive behaviour was measured during the photophase and scotophase immediately before injection of BCG as shown in Figure 5. The values are means ± SEM, *n* = 9.

Injection of BCG caused long-term changes in the circadian patterns of locomotion (Figure 5A) and exploration (Figure 5B), which in NPY−/−;PYY−/− mice were more pronounced than in WT mice. Characteristically, the circadian amplitude of locomotion and exploration was greatly attenuated by BCG due to a reduction of nocturnal activity. Although WT mice had considerably recovered from the BCG-induced suppression of circadian locomotion by 7 days post-injection, the amplitude of circadian locomotion in NPY−/−;PYY−/− mice stayed attenuated throughout the 15 day recording period after BCG injection (Figure 5A). The same was true for the circadian pattern of exploration that in NPY−/−;PYY−/− mice was practically abolished for several days post-injection (Figure 5B). BCG also reduced the circadian pattern of exploration in WT animals to a considerable extent, and this effect likewise failed to reverse completely during the 15 day post-injection recording period (Figure 5B).

The effect of BCG on the circadian patterns of drinking and feeding in WT and NPY−/−;PYY−/− animals is illustrated in Figure 5C and D. BCG caused a transient reduction of the circadian amplitude of water and food intake, which in WT animals reversed faster than in NPY−/−;PYY−/− mice (Figure 5C and D). It is worth noting, however, that even in NPY−/−;PYY−/− animals, both parameters of ingestion recovered completely during the 15 day post-injection recording period.

**Figure 6:**
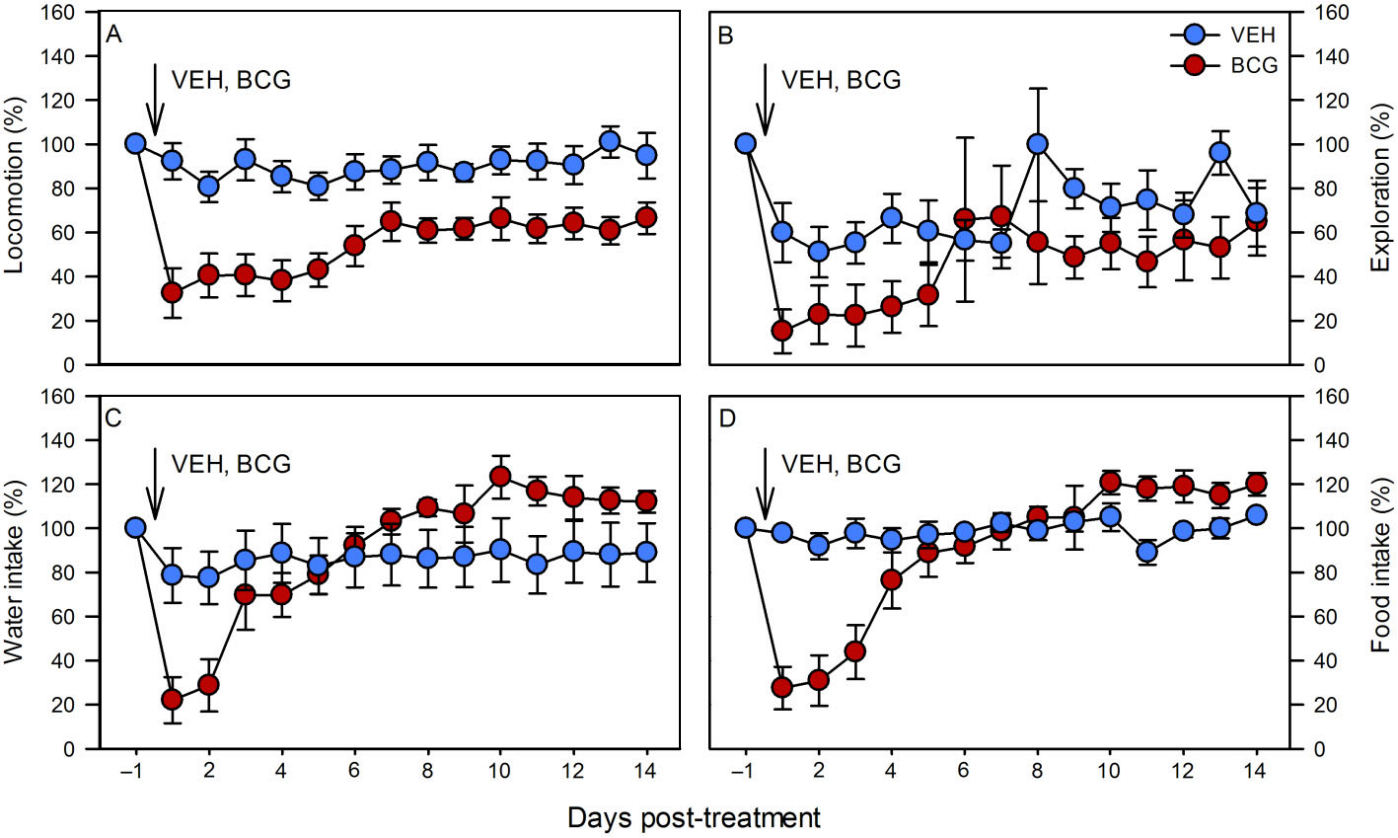
Effect of BCG (10^8^ CFU per mouse injected i.p.), relative to VEH, on the daily levels of locomotion (A), exploration (B), drinking (C) and feeding (D) in male NPY−/−;PYY−/− mice. VEH or BCG was injected as indicated. The graphs show the daily levels of activity for 1 day before (set as 100%) and 14 days after BCG injection. The values are means ± SEM, *n* = 6 (VEH) and 9 (BCG).

**Figure 7:**
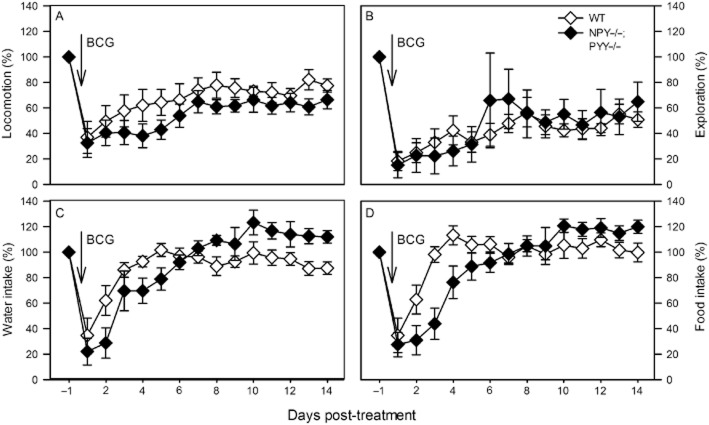
Effect of BCG (10^8^ CFU per mouse injected i.p.) on the daily levels of locomotion (A), exploration (B), drinking (C) and feeding (D) in male WT and NPY−/−;PYY−/− mice. BCG was injected as indicated. The graphs show the original recordings depicted in Figure 5A–D in a normalized manner: the daily levels of activity recorded after BCG injection are expressed as a percentage of the activity recorded 1 day before BCG injection (set as 100%). The values are means ± SEM, *n* = 9.

Quantitative figures of locomotion, exploration and ingestion suitable for statistical evaluation were obtained by summing up the activity scores recorded during the photo-and scotophase of each day and presenting them as daily activity scores (Figures 6 and 7). In order to account for the genotype-related differences in the basal circadian patterns of behaviour, the daily activity scores recorded during the day before injection were set as 100%, and the daily activity scores measured post-injection expressed as a percentage of the pre-injection score. The statistical analysis of the data addressed both the maximal effects of BCG, relative to VEH, and their time course. Figure 6A–D compares the effect of VEH and BCG on the daily activities in NPY−/−;PYY−/− mice. Although locomotion, drinking and feeding were *grosso modo* left unchanged by VEH, exploration was reduced by VEH for 7 days (Figure 6B). Relative to VEH, BCG caused a statistically significant attenuation of locomotion, exploration, drinking and feeding (Figure 6A–D; Table 2). Compared with the effect of VEH, the effect of BCG on exploration, drinking and feeding had waned within 4–6 days post-injection (Figure 6B–D), whereas the effect on locomotion did not fully reverse within the 15 day post-injection recording period (Figure 6A). Two-way anova for repeated measurements showed that locomotion varied with time [*F*_(14,182)_ = 11.61, *P* < 0.01] and treatment [*F*_(1,13)_ = 11.46, *P* < 0.01] with a significant interaction between these factors [*F*_(14,182)_ = 5.36, *P* < 0.01]. Exploration differed with time [*F*_(14,182)_ = 4.53, *P* < 0.01] but not treatment [*F*_(1,13)_ = 1.68, *P* = 0.22], without a significant interaction between these factors [*F*_(14,182)_ = 1.45, *P* = 0.13]. Water intake depended on time [*F*_(14,182)_ = 10.48, *P* < 0.01] but not treatment [*F*_(1,13)_ = 0.12, *P* = 0.74], with a significant interaction between these factors [*F*_(14,182)_ = 6.87, *P* < 0.01]. Likewise, food intake varied with time [*F*_(14,182)_ = 11.12, *P* < 0.01] but not treatment [*F*_(1,13)_ = 0.18, *P* = 0.21], with a significant interaction between these factors [*F*_(14,182)_ = 8.84, *P* < 0.01].

**Table 2:** Effect of BCG, relative to VEH, to decrease daily locomotion, exploration, drinking and feeding in NPY−/−;PYY−/− mice

Treatment	Parameter	Value	*P*
VEH	Daily locomotion (%)	80.67 ± 6.93	
BCG	Daily locomotion (%)	32.49 ± 11.29	<0.01
VEH	Daily exploration (%)	51.08 ± 11.44	
BCG	Daily exploration (%)	15.19 ± 9.95	=0.04
VEH	Daily drinking (%)	77.49 ± 11.88	
BCG	Daily drinking (%)	22.06 ± 10.49	<0.01
VEH	Daily feeding (%)	89.09 ± 5.55	
BCG	Daily feeding (%)	27.54 ± 9.62	<0.01

The table shows the maximal effects of VEH and BCG (10^8^ CFU per mouse injected i.p.) to decrease the daily levels of locomotion, exploration, drinking and feeding. The levels of activity are expressed as a percentage of the values measured before BCG injection (set as 100%). The values are means ± SEM, *n* = 6 (VEH) and 9 (BCG).

The daily activities of locomotion, exploration and ingestion in WT and NPY−/−;PYY−/− animals, shown in Figure 5A–D, are presented in Figure 7A–D in a normalized manner, because the basal activities differ in the two genotypes. Although the maximal effect of BCG to attenuate locomotion, exploration, drinking and feeding did not significantly differ between WT and NPY−/−;PYY−/− mice (Figure 7A–D; Table 3), there were differences in the recovery rate. As can be seen in Figure 7C and D, the BCG-evoked depression of drinking and feeding reversed in both genotypes within 5 days post-injection, albeit with an appreciable delay in NPY−/−;PYY−/− mice. In contrast, the BCG-evoked depression of locomotion and exploration failed to fully reverse in either genotype during the recording period (Figure 7A and B). Two-way anova for repeated measurements showed that locomotion varied with time [*F*_(14,210)_ = 17.00, *P* < 0.01] but not genotype [*F*_(1,15)_ = 1.51, *P* = 0.24], without a significant interaction between these factors [*F*_(14,210)_ = 0.85, *P* = 0.61]. Exploration also differed with time [*F*_(14,210)_ = 8.49, *P* < 0.01] but not genotype [*F*_(1,15)_ = 0.64, *P* = 0.80], without a significant interaction between these factors [*F*_(14,210)_ = 0.216, *P* = 0.76]. Water intake depended on time [*F*_(14,224)_ = 20.61, *P* < 0.01] but not genotype [*F*_(1,15)_ = 0.245, *P* = 0.63], with a significant interaction between these factors [*F*_(14,224)_ = 3.97, *P* < 0.01]. Likewise, food intake varied with time [*F*_(14,224)_ = 20.81, *P* < 0.01] but not genotype [*F*_(1,15)_ = 1.00, *P* = 0.33], with a significant interaction between these factors [*F*_(14,224)_ = 4.08, *P* < 0.01].

**Table 3:** Effect of BCG to decrease daily locomotion, exploration, drinking and feeding in WT and NPY−/−;PYY−/− mice

Genotype	Parameter	Value	*P*
WT	Daily locomotion (%)	36.89 ± 12.55	
NPY−/−;PYY−/−	Daily locomotion (%)	32.49 ± 11.29	=0.80
WT	Daily exploration (%)	18.32 ± 7.60	
NPY−/−;PYY−/−	Daily exploration (%)	15.19 ± 9.95	=0.81
WT	Daily drinking (%)	34.73 ± 13.50	
NPY−/−;PYY−/−	Daily drinking (%)	22.06 ± 10.49	=0.47
WT	Daily feeding (%)	34.68 ± 13.56	
NPY−/−;PYY−/−	Daily feeding (%)	27.54 ± 9.62	=0.67

The table shows the maximal effect of BCG (10^8^ CFU per mouse injected i.p.) to decrease the daily levels of locomotion, exploration, drinking and feeding. The levels of activity are expressed as a percentage of the values measured before BCG injection (set as 100%). The values are means ± SEM, *n* = 9.

## Discussion

### Rationale of the study

BCG (Connaught strain) is an attenuated strain of *Mycobacterium bovis* that in freeze-dried viable preparations has long been used as tuberculosis vaccine and anti-neoplastic drug. Due to stimulation of the innate immune system, it can evoke local and systemic inflammatory reactions including fever and, at high doses, septic shock (Allie *et al*., 2008; Moreau *et al*., 2008; O'Connor *et al*., 2009a). Injected i.p. in mice, BCG has been found to elicit a systemic immune response characterized by an increase in circulating cytokines (Sander *et al*., 1995; Leal *et al*., 2001) and behavioural symptoms of sickness behaviour (Moreau *et al*., 2008). The sickness response to immune challenge is related to the induction of pro-inflammatory cytokines, including IL-6, which signal to the brain via a neural and endocrine route (Goehler *et al*., 2000; Dantzer *et al*., 2008; Gibb *et al*., 2008; Haroon *et al*., 2012). The acute reaction to BCG is followed by a period of depression-like behaviour that involves cytokine induction and indoleamine 2,3-dioxygenase up-regulation, whereas the acute sickness response takes place independently of these factors (Moreau *et al*., 2008; O'Connor *et al*., 2009a,b2009b).

The present study therefore aimed at shedding more light on the factors that regulate the initial biological response to BCG. Used instead of LPS, BCG offers the advantage of studying the sickness response to a true infection by a living bacterium instead of addressing the reaction to a bacterial cell wall constituent. Relative to LPS, BCG causes a more pronounced stimulation of the innate immune system because it activates an array of different pattern recognition receptors, which is reflected by a prolonged immune and sickness response (Moreau *et al*., 2008). Because the NPY system has been shown to protect against the behavioural responses to LPS (Painsipp *et al*., 2008; 2010,), we set out to explore whether this role of the NPY system also pertains to some of the biological effects of BCG.

### BCG causes prolonged increase of circulating corticosterone and anxiety in C57BL/6 mice

The role of the NPY system in the sickness response to BCG was investigated by comparative phenotyping of WT, PYY−/−, NPY−/− and NPY−/−;PYY−/− mice, which have a C57BL/6:129/SvJ (1:1) background. Using C57BL/6 and C57BL/6–129/SvJ (WT) mice and a BCG dose of 1 × 10^8^ CFU per mouse (O'Connor *et al*., 2009a), we were able to reproduce key components of the systemic response to BCG, which previously has been studied in CD1 mice (Moreau *et al*., 2008; O'Connor *et al*., 2009a,b2009b). Besides causing non-significant fever and transient weight loss, BCG elevated the plasma level of corticosterone as estimated 1 day post-treatment. This finding indicates that BCG causes a prolonged stimulation of the HPA axis that outlasts the duration of stress-induced corticosterone release, which in rodents normalizes within 2–3 h (Vázquez, 1998; Brabham *et al*., 2000; Célérier *et al*., 2004). The prolonged stimulation of the HPA axis may also have a bearing on the delayed increase in anxiety (McEwen, 2007) as observed 7 days post-BCG.

### NPY and PYY protect against BCG-induced weight loss and metabolic dysbalance

Having confirmed that BCG induces a sickness response in C57BL/6 and WT mice as much as it does in CD1 mice, we discovered that NPY and PYY play a role in regulating the biological responses to BCG. An important outcome of the pertinent experiments was that knockout of PYY and NPY, alone and in combination, has a differential effect on various elements of the systemic BCG reaction. The BCG-induced weight loss was aggravated by knockout of either peptide and particularly pronounced by combined deletion of NPY and PYY. Thus, NPY−/−;PYY−/− mice lost some 15% of weight that failed to recover significantly within 15 days post-BCG treatment. These observations need to be seen in context with the implication of the NPY/PYY system in regulating food intake, BW and metabolic homeostasis (Erickson *et al*., 1996; Gehlert, 1999; Herzog, 2003; McGowan and Bloom, 2004; Ueno *et al*., 2008). Although the BW remains unaltered by knockout of NPY (Erickson *et al*., 1996; Lin *et al*., 2004; Edelsbrunner *et al*., 2009), male PYY−/− mice weigh more than WT mice (Edelsbrunner *et al*., 2009). Peptide knockout experiments also indicate that NPY and PYY stimulate food intake in order to meet the energy demand that they generate by enforcing locomotor and exploratory activity (Edelsbrunner *et al*., 2009). NPY appears to play a greater role than PYY in controlling ingestion, activity and BW because the phenotypic overlap between NPY−/− and NPY−/−;PYY−/− mice is closer than that between PYY−/− and NPY−/−;PYY−/− mice (Edelsbrunner *et al*., 2009). In addition, NPY may mediate some of the biological actions of PYY as has been suggested for the effect of PYY on insulin secretion and fasting-induced hyperphagia (Zhang *et al*., 2012). The implication of NPY and PYY in controlling circadian activity (Harrington *et al*., 2007; Edelsbrunner *et al*., 2009) was also borne out in the present study in which the amplitude of the circadian locomotor and exploratory behaviour was attenuated in NPY−/−;PYY−/− mice, relative to WT animals. NPY has previously been found to modify the setting of the circadian clock and contribute to the maintenance of the circadian pattern of locomotor activity (Harrington *et al*., 2007).

### The protective effect of NPY against BCG-induced weight loss does not involve inhibition of BCG-evoked IL-6 formation

The prolonged weight loss caused by BCG is in all likelihood related to the prolonged effect of BCG to stimulate the immune system (Moreau *et al*., 2008). This was confirmed in the present study in which the circulating levels of IL-6 remained elevated 16 days post-BCG treatment. IL-6 is thought to play a priming role in the early phase of the immune response to BCG (Huygen *et al*., 1991; Sander *et al*., 1995; Leal *et al*., 2001), yet the current observations indicate that IL-6, like IFN-γ (Moreau *et al*., 2008), also represents an index of the long-term action of BCG on the immune system.

However, the protective effect of NPY against the BCG-induced weight loss does not arise from inhibition of BCG-evoked IL-6 formation. This possibility was envisaged because NPY is known to exert a regulatory role on innate and adaptive immune function (Wheway *et al*., 2007). Because the deletion of PYY, NPY or NPY plus PYY failed to significantly alter the effect of BCG to cause a prolonged rise of circulating IL-6 as measured 16 days post-injection, we conclude that NPY counteracts the BCG-evoked weight loss at a level beyond immune stimulation.

The site of action of NPY and PYY to protect from the BCG-evoked weight loss cannot be deduced from the current experiments. It is conceivable that this action takes place either within or outside the CNS. The latter possibility can be envisaged from findings in the rat in which immune stimulation by LPS causes skeletal muscle atrophy by an effect that involves the HPA axis (Braun *et al*., 2011) and the sympathetic nervous system (Braun and Marks, 2011). It awaits to be examined whether NPY expressed by sympathetic neurons (Hirsch and Zukowska, 2012) plays a role in this effect.

### The protective effect of NPY against BCG-induced weight loss is at variance with its effect on ingestive behaviour

The finding that the BCG-evoked loss of BW was most pronounced in NPY−/−;PYY−/− mice ascribes both peptides an important role in maintaining metabolic homeostasis in the face of immune challenge. Further analysis of this effect in the LabMaster system revealed that the reduction of BW, which in NPY−/−;PYY−/− mice persisted for the whole observation period of 15 days, cannot conclusively be explained by alterations of activity and food intake. Thus, the BCG-induced inhibition of drinking and feeding in NPY−/−;PYY−/− mice had reversed in less than 1 week post-injection, whereas the BCG-induced suppression of locomotion and exploration failed to fully reverse during the 15 day observation period in WT and NPY−/−;PYY−/− mice alike (Figures 6 and 7). In contrast, BW failed to significantly recover although energy intake (feeding) had returned to normal while, as judged from the locomotor and exploratory activity, energy expenditure stayed low.

It follows that the pronounced and prolonged weight loss that BCG induced in NPY−/−;PYY−/− mice was related to a disturbance of metabolic homeostasis. In other terms, NPY and PYY protect against the prolonged effect of BCG-evoked immune challenge on weight loss and metabolic dysbalance, but not against the hypophagic effect of BCG. Combined deletion of PYY and NPY retards, however, the recovery from the hypophagic effect of BCG. To the contrary, hyperphagia provoked by food deprivation is enhanced in PYY−/− animals but left unchanged in NPY−/− as well as NPY−/−;PYY−/− mice (Zhang *et al*., 2012), although another study holds that fasting-induced hyperphagia is also enhanced in NPY−/− animals (Erickson *et al*., 1996). These observations suggest that the reduction of food intake caused by fasting, on the one hand, and BCG, on the other hand, involves the NPY/PYY system to a different extent. Furthermore, the present findings indicate that the NPY/PYY system plays different roles in the anorectic and anti-dipsogenic effects of immune challenge by LPS (Edelsbrunner *et al*., 2009) and BCG.

## Conclusions

The current study demonstrates that NPY and, to some extent, PYY participates in the ability of immune challenge with BCG to cause prolonged weight loss and metabolic dysbalance. It is not possible to deduce conclusively from the current results whether these effects of NPY and PYY take place in the periphery and/or in the cerebral circuitry involved in the processing of the sickness response and related biological reactions to immune challenge. Importantly, NPY has the potential to affect both innate and adaptive immunity, leading to either immune activation or suppression depending on its concentration, the Y receptors activated and cell types involved (Wheway *et al*., 2007). Although it must not be neglected that developmental compensations may mask the full implication of PYY and NPY, our results attest to an important physiological role of NPY and PYY in orchestrating multiple homeostatic reactions in the face of infection and immune stimulation. Previous experiments addressing the acute and delayed behavioural reactions to LPS have indicated that both Y2 and Y4 receptors play a role in preventing the adverse impact of LPS on the brain (Painsipp *et al*., 2008; 2010,). Detailed analysis of the receptors and signalling pathways involved in the effects of NPY and PYY on the immune-brain axis may help identifying novel pharmacological targets for the management of immune-related metabolic diseases.

## Abbreviations

BCG: Bacille Calmette–Guérin
BW: body weight
HPA: hypothalamic-pituitary-adrenal
NPY: neuropeptide Y
PYY: peptide YY
VEH: vehicle
WT: wild type
